# Viruses of Haloarchaea

**DOI:** 10.3390/life4040681

**Published:** 2014-11-13

**Authors:** Alison W. S. Luk, Timothy J. Williams, Susanne Erdmann, R. Thane Papke, Ricardo Cavicchioli

**Affiliations:** 1 School of Biotechnology and Biomolecular Sciences, University of New South Wales, Sydney, New South Wales 2052, Australia; E-Mails: alisonluk@gmail.com (A.W.S.L.); t.williams@unsw.edu.au (T.J.W.); s.erdmann@unsw.edu.au (S.E.); 2 Department of Microbiology, The Chinese University of Hong Kong, Sha Tin, Hong Kong, China; 3 Department of Molecular and Cell Biology, University of Connecticut, Storrs, CT 06269-3125, USA; E-Mail: robertson.papke@uconn.edu

**Keywords:** viral lineage, viral evolution, virus life cycle, capsid protein, persistent, temperate, virulent infection, CRISPR, host defense, evasion invasion mechanism, integrase, genome variation, salty, halophile

## Abstract

In hypersaline environments, haloarchaea (halophilic members of the *Archaea*) are the dominant organisms, and the viruses that infect them, haloarchaeoviruses are at least ten times more abundant. Since their discovery in 1974, described haloarchaeoviruses include head-tailed, pleomorphic, spherical and spindle-shaped morphologies, representing *Myoviridae*, *Siphoviridae*, *Podoviridae*, *Pleolipoviridae*, *Sphaerolipoviridae* and *Fuselloviridae* families. This review overviews current knowledge of haloarchaeoviruses, providing information about classification, morphotypes, macromolecules, life cycles, genetic manipulation and gene regulation, and host-virus responses. In so doing, the review incorporates knowledge from laboratory studies of isolated viruses, field-based studies of environmental samples, and both genomic and metagenomic analyses of haloarchaeoviruses. What emerges is that some haloarchaeoviruses possess unique morphological and life cycle properties, while others share features with other viruses (e.g., bacteriophages). Their interactions with hosts influence community structure and evolution of populations that exist in hypersaline environments as diverse as seawater evaporation ponds, to hot desert or Antarctic lakes. The discoveries of their wide-ranging and important roles in the ecology and evolution of hypersaline communities serves as a strong motivator for future investigations of both laboratory-model and environmental systems.

## 1. Introduction

Viruses infect all three domains of life, with viruses of *Archaea* being the least studied. Currently, around 100 viruses infecting *Archaea* (archaeoviruses) have been described, compared to around 6200 bacteriophages [1,2,3]. Nevertheless, research into archaeoviruses has increased in recent years, and in the same ways that studies of *Archaea* uncovered unique traits about their cellular adaptation, ecology and evolution [4], there is no doubt that archaeoviruses are engendering high levels of interest.

Haloarchaea are members of the *Archaea* that live in hypersaline environments ranging from 10% salinity to salt saturation (~36% salinity) [5]. Haloarchaea are found in a wide range of environments that differ in their geography, climate, limnology and chemistry, and include salt lakes, soda lakes and artificially formed seawater evaporation ponds. Haloarchaea tend to be the dominant cellular forms in hypersaline environments above 15% NaCl, with halophilic bacteria typically contributing less than a quarter of the population [2,6,7]. *Eucarya* tend to be even less abundant, but may be diverse [8,9,10], with the phototrophic green alga, *Dunaliella* spp. being an important primary-producer and source of nutrients [2,6,7]. A general feature of hypersaline environments is that the communities tend to have low complexity. Relative to other aquatic systems, they can also sustain high concentrations of viruses, with virus-like particles per mL reaching up to 1.3 × 10^10^ [11,12].

Viruses infecting haloarchaea, haloarchaeoviruses [1,13], were first discovered in 1974, several years before *Archaea* were described as a lineage of life distinct from *Bacteria* and *Eucarya* [4,14,15]. By 2003, fifteen haloarchaeoviruses were described, and methods for the isolation and cultivation of haloarchaeoviruses were published in 2006 [16,17]. In 2012, two large studies on haloarchaeoviruses were performed, one culture-dependent [2], the other culture-independent [12], contributing to a total of 33 haloarchaeoviruses, and another 34 putative haloarchaeoviruses which either are not yet genome sequenced, or were constructed from metaviromes, but not yet confirmed with laboratory isolation.

Forty years since the first discovery of haloarchaeoviruses, relatively few have been investigated in great detail, and many studies have been discontinued after initial attempts [6,18]. This is due in part to difficulties in isolating and cultivating haloarchaea hosts, and to date, the majority of research performed on haloarchaeoviruses has been with strains of *Halorubrum* spp. and *Haloarcula* spp., which tend to be amenable to laboratory manipulation.

## 2. Classification

In hypersaline environments, virus-like particles with a variety of different morphotypes have been described, including head-tailed, spindle-shaped, spherical and filamentous, ranging from 40 to 100 nm in diameter [11,19,20,21,22]. During microscopy examinations, filamentous forms may be interpreted as being bacteriophages because this morphology has not been described for many archaeoviruses, an exception being the prevalence of filamentous forms that infect thermophilic *Archaea* [23]. In some hypersaline environments, spherical forms are the most abundant, with spindle-shaped forms increasing in abundance with increasing salinity [6,11]. In environments close to salt saturation, spindle-shaped virus-like particles appear to be the most abundant, followed by spherical forms, with head-tailed forms representing a minority of up to 1% in some systems [19,21,22].

Traditionally, viruses/phage have been classified into only two modes of infection: virulent and temperate. Virulent viruses perform a lytic cycle and form progeny within their host after infection, and do not form stable lysogens with their hosts. Temperate viruses do have the capacity to form stable lysogens with their hosts, in addition to being able to undergo a lytic cycle. This classification is inadequate to describe all haloarchaeoviruses, as many are more aptly described as having persistent infection. Persistent infection differs from temperate infection by host cells not containing viral DNA in a provirus form (*i.e.*, integrated in the host chromosome) [24]. Persistent infection also differs from virulent infection by viruses forming unstable carrier states and continuously producing and releasing progeny at a low rate without causing host cell lysis [25,26,27].

For all cellular life, taxonomic classification and phylogeny can be inferred by comparing universally conserved marker genes, typically small subunit ribosomal RNA genes. However, as universal marker genes are not present in viruses, inferring viral lineages is inherently more difficult. Below we briefly consider how viral lineages can be interpreted, in order to rationalize a satisfactory means of classifying haloarchaeoviruses.

### Viral Lineages

Haloarchaeoviruses that infect taxonomically diverse hosts have viral capsids with very similar structures and protein motifs, but do not have many detectable genetic sequence similarities. One explanation is that while there is scope to diversify gene sequence and gene content, there are only a limited number of plausible structures for viruses, and therefore there is a structural convergence of virus form [28,29]. The lack of sequence identity would be consistent with a polyphyletic origin for viruses [28]. If certain lineages of viruses existed before the last universal common ancestor of cellular life, viruses could have diverged to evolve with distinct host types [30,31]. Certain genes, such as structural and assembly genes, could be vertically inherited if strong evolutionary constraints restrict their divergence. If these genes are conserved, viruses infecting different hosts would have similar structures. In comparison, genes for replication, host interaction and virus release could have less evolutionary constraint, thereby enabling recombination events to be more readily inherited and detected more frequently than for non-structural genes. A relatively high level of permissive recombination for these genes would lead to the ability of viruses to evolve and maintain effective interactions with hosts.

Because all viruses do not share a universal marker gene, viruses have traditionally been classified based on their genome types, sequence similarities and general morphology. However, genomic similarity may not provide the best measure of phylogeny [28], or inference of host type [32]. Viral communities may represent a pool of DNA that overlaps and can be exchanged with their hosts, thereby underpinning a phylogenetic web of lateral and vertical inheritance, rather than supporting a coherent virus phylogenetic tree [33,34]. Therefore traditional classification may not accurately define the evolutionary relationships of viruses or be suitable for assigning viruses to higher order taxa [29,35,36]. Instead, it has been argued that comparison of coat protein structures and virion architecture may be better for approximating phylogenetic classification of viruses, with a structure-based determination of viral lineages applying universally to viruses infecting different domains of life [29,32,36,37].

Support for the validity of this type of classification comes from the viral lineage proposed for head-tailed viruses (caudoviruses) containing conserved Hong Kong 97 (HK97)-like main capsid proteins [38]. The HK97 protein fold was found in viruses infecting all three domains, including seven bacteriophages, the eucaryovirus herpes simplex virus type 1, and recently haloarchaeovirus HSTV-1 [38]. Within the *Archaea*, a viral lineage has been proposed that encompasses all short-tailed spindle-shaped viruses, which infect both archaeal kingdoms *Crenarchaeota* and *Euryarchaeota* [39].

A second line of support relates to the proposed vertical β-barrel superlineage of viruses [30,40]. This superlineage comprises of two sublineages; PRD1-type viruses with double β-barrel capsid proteins which infect all three domains of life, and halosphaerovirus SH1 and bacteriophage P23-77 with single β-barrel capsid proteins [30,40,41,42,43]. If viruses are restricted by structural constraints, there is likely to be a limited number of unique viral coat proteins, and therefore a limited number of structure-based viral lineages to be discovered [29].

**Figure 1:**
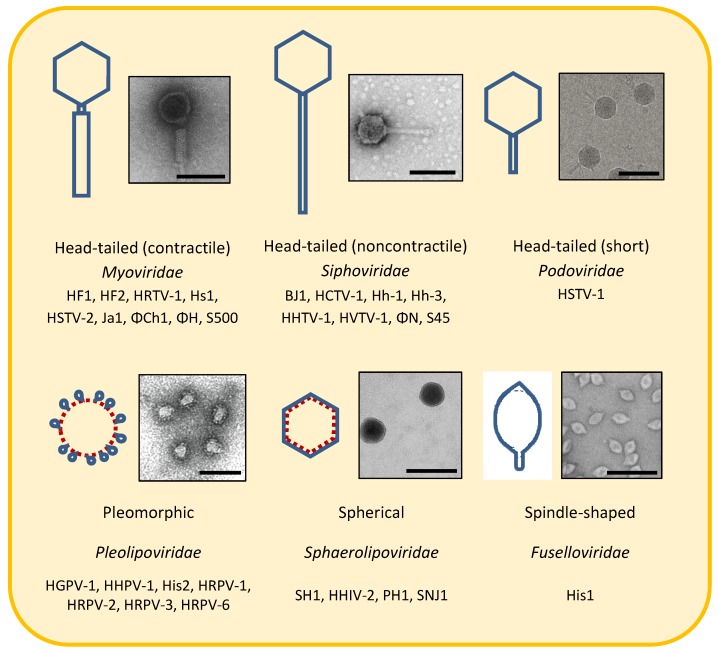
Morphotypes of haloarchaeoviruses. Blue lines represent protein and red dotted lines represent lipids. Proposed viral families are included. Note that *Pleolipoviridae* and *Sphaerolipoviridae* families, and the classification of many haloarchaeoviruses into existing families, have not yet been formally accepted by the International Committee on Taxonomy of Viruses. Haloarchaeoviruses of each morphotype are listed except for head-tailed viruses with undetermined tail morphologies. Electron micrographs show isolates representing each morphotype, head-tailed (contractile) ΦCh1 [51]; head-tailed (non-contractile) BJ1 [63]; head-tailed (short) HSTV-1 [38]; pleomorphic HRPV-1 [69]; spherical SH1 [17]; spindle-shaped His1 [39]. Scale bars represent 100 nm.

Archaeovirus morphologies have previously been reviewed and classified into eight morphotypes, with 15 families described by 2012 [1,3]. In this review, described haloarchaeoviruses are classified into four morphotypes (Table 1), and many are classified into six families (Figure 1). While it is impossible to know if all extant viruses had a common ancestor, it seems reasonable to classify haloarchaeoviruses based on viral lineages that incorporate subdivisions based primarily on morphology and virion architecture, with genomic information being used to refine classification (e.g.,further subdivision).

**Table 1:** Characteristics of haloarchaeoviruses (adapted from [44]).

Virus ^1^	Host ^2^	Mode of Infection ^3^	Source	Morphology ^4^	Size/nm	Genome Type	Genome Size/kb	G + C mol%	p*I*	References
HATV-1	*Haloarcula* sp.	Virulent	Saltern, Thailand	Head-tailed (contractile)	-	-	-	-	-	[2]
HATV-2	*Har.* sp., *Halorubrum.* sp.	Virulent	Saltern, Israel	Head-tailed (contractile)	-	-	-	-	-	[2]
HF1	*Halobacterium salinarum, Haloferax volcanii*	Persistent	Saltern, Australia	Head-tailed (contractile)	Head 67.8 ± 3, Tail 90 ± 2	Linear dsDNA	75.9	55.8	<5	[25,45]
HF2	*Halorubrum coriense, Halorubrum saccharovorum*	Persistent	Saltern, Australia	Head-tailed (contractile)	Head 58, Tail 94	Linear dsDNA	77.7	55.8	<5	[25,45,46]
HGTV-1	*Halogranum* sp.	Virulent	Saltern, Thailand	Head-tailed (contractile)	-	Linear dsDNA	143.9	50.4	-	[2,47]
HJTV-1	*Haloarcula japonica*	Virulent	Saltern, Italy	Head-tailed (contractile)	-	-	-	-	-	[2]
HJTV-2	*Har. Japonica*	Virulent	Saltern, Thailand	Head-tailed (contractile)	-	-	-	-	-	[2]
HRTV-1	*Hrr.* sp.	-	Saltern, Italy	Head-tailed (contractile)	Head 50, Tail 87	-	-	-	-	[48]
HRTV-2	*Hrr.* sp.	Virulent	Saltern, Italy	Head-tailed (contractile)	-	-	-	-	-	[2]
HRTV-3	*Hrr.* sp.	Virulent	Saltern, Italy	Head-tailed (contractile)	-	-	-	-	-	[2]
HRTV-5	*Hrr.* sp.	Virulent	Saltern, Italy	Head-tailed (contractile)	-	Linear dsDNA	76.1	56.4	-	[2,47]
HRTV-6	*Hrr.* sp.	Virulent	Saltern, Italy	Head-tailed (contractile)	-	-	-	-	-	[2]
HRTV-7	*Hrr.* sp.	Virulent	Saltern, Italy	Head-tailed (contractile)	-	Linear dsDNA	69.0	59.6	-	[2,47]
HRTV-8	*Hrr.* sp.	Virulent	Saltern, Thailand	Head-tailed (contractile)	-	Linear dsDNA	74.5	57.1	-	[2,47]
HRTV-9	*Hrr.* sp*.*	Virulent	Saltern, Israel	Head-tailed (contractile)	-	-	-	-	-	[2]
HRTV-10	*Hrr.* sp*.*	Virulent	Saltern, Israel	Head-tailed (contractile)	-	-	-	-	-	[2]
HRTV-11	*Hrr.* sp*.*	Virulent	Saltern, Israel	Head-tailed (contractile)	-	-	-	-	-	[2]
HRTV-12	*Hrr.* sp*.*	Virulent	Saltern, Spain	Head-tailed (contractile)	-	-	-	-	-	[2]
Hs1	*Hbt. Salinarum*	Persistent	*Hbt. salinarum* cultures from salted codfish	Head-tailed (contractile)	Head 50, Tail 120	-	-	-	-	[14,49]
HSTV-2	*Halorubrum. Sodomense*	Virulent	Saltern, Israel	Head-tailed (contractile)	Head 60, Tail 101 ± 5	Linear dsDNA	68.2	60.0	-	[2,50]
HSTV-3	*Hrr. sodomense*	Virulent	Saltern, Israel	Head-tailed (contractile)	-	-	-	-	-	[2]
Ja1	*Hbt. Salinarum*	Virulent	Salt ponds, Jamaica	Head-tailed (contractile)	Head 90, Tail 150	-	-	-	-	[5]
ΦCh1	*Natrialba magadii*	Temperate	*Nab. magadii* culture	Head-tailed (contractile)	Head 70, Tail 130	Linear dsDNA	58.5	61.9	<5.2	[51,52,53]
ΦH	*Hbt. Salinarum*	Temperate	*Hbt. salinarum* culture	Head-tailed (contractile)	Head 64, Tail 170	Linear dsDNA	59.0	65.0	-	[54,55,56,57,58,59,60,61]
S5100	*Hbt. salinarum*	Persistent	Salt ponds, Jamaica	Head-tailed (contractile)	-	dsDNA	-	-	-	[62]
BJ1	*Hrr.* sp.	Temperate	Salt lake, Mongolia	Head-tailed (non-contractile)	Head 56, Tail 71	Linear dsDNA	42.3	64.0	<5	[63]
HCTV-1	*Haloarcula californiae*	Virulent	Saltern, Italy	Head-tailed (non-contractile)	Head 63, Tail 80	Linear dsDNA	103.2	57.0	-	[47,48]
HCTV-2	*Har. californiae*	Virulent	Saltern, Thailand	Head-tailed (non-contractile)	-	Linear dsDNA	54.3	68.1	-	[2,47]
HCTV-5	*Har. californiae*	Virulent	Saltern, Thailand	Head-tailed (non-contractile)	-	Linear dsDNA	102.1	57.6	-	[2,47]
Hh-1	*Hbt. salinarum*	Persistent	Anchovy sauce, Philippines	Head-tailed (non-contractile)	Head 60, Tail 100	dsDNA	32.7	67.1	-	[64,65]
Hh-3	*Hbt. salinarum*	Persistent	Anchovy sauce, Philippines	Head-tailed (non-contractile)	Head 75, Tail 50	dsDNA	29.4	62.2	-	[64,65]
HHTV-1	*Haloarcula hispanica*	Virulent	Saltern, Italy	Head-tailed (non-contractile)	Head 50, Tail 110	Linear dsDNA	49.1	56.5	-	[47,48]
HHTV-2	*Har. hispanica*	Virulent	Saltern, Thailand	Head-tailed (non-contractile)	-	Linear dsDNA	52.6	66.6	-	[2,47]
HRTV-4	*Hrr.* sp.	Virulent	Saltern, Italy	Head-tailed (non-contractile)	-	Linear dsDNA	35.7	59.5	-	[2,47]
HVTV-1	*Haloarcula vallismortis*	Virulent	Saltern, Thailand	Head-tailed (non-contractile)	Head 70, Tail 73 ± 5	Linear dsDNA	101.7	58.0	-	[2,50]
ΦN	*Hbt. salinarum*	Virulent	*Hbt. salinarum* NRL/JW cultures	Head-tailed (non-contractile)	Head 55, Tail 85	Linear dsDNA	56.0	70.0	-	[66]
S45	*Hbt. salinarum*	Virulent	Salt ponds, Jamaica	Head-tailed (non-contractile)	Head 40, Tail 70	dsDNA	-	-	-	[67]
HSTV-1	*Haloarcula sinaiiensis, Har. vallismortis*	Virulent	Saltern, Italy	Head-tailed (short)	Head 60, Tail 40	Circular dsDNA	32.2	60.0	-	[2,38]
EHP-2	Putative *Haloquadratum walsbyi*	-	Saltern, Spain	Putative head-tailed	-	Linear dsDNA	27.2	43.9	-	[12]
EHP-5							29.5	44.1		
EHP-9							30.1	45.8		
EHP-22							33.8	43.8		
EHP-24							32.7	44.2		
EHP-29							21.5	44.4		
EHP-37							30.3	44.8		
EHP-38							26.6	44.6		
EHP-39							21.3	44.0		
EHP-40							33.5	44.1		
EHP-41							20.2	44.8		
EHP-42							23.1	44.9		
EHP-D7							31.1	44.8		
EHP-E5							32.7	45.0		
EPH-11	Putative *Halorubrum lacusprofundi*	-	Saltern, Spain	Putative head-tailed	-	Linear dsDNA	33.5	58.5	-	[12]
EPH-14							20.2	57.8		
EPH-32							34.4	60.4		
HGPV-1	*Halogeometricum* sp.	Persistent	Saltern, Spain	Pleomorphic	55.5 ± 5.2	Circular dsDNA	9.7	61.6	-	[2,26,68]
HHPV-1	*Har. hispanica*	Persistent	Saltern, Italy	Pleomorphic	51.7 ± 4.0	Circular dsDNA	8.1	55.8	-	[26,35,48,69]
His2	*Har. hispanica*	Persistent	Salt lake, Australia	Pleomorphic	70.6 ± 3.6	Linear dsDNA	16.1	39.0–40.0	<7	[24,26,70]
HRPV-1	*Hrr.* sp.	Persistent	Saltern, Italy	Pleomorphic	41.1 ± 2.2	Circular ssDNA	7.0	54.2	-	[26,69]
HRPV-2	*Hrr.* sp.	Persistent	Saltern, Thailand	Pleomorphic	54.0 ± 4.3	ssDNA	10.7	63.7	-	[2,26]
HRPV-3	*Hrr.* sp.	Persistent	Artificial salt pond, Israel	Pleomorphic	67.2 ± 5.2	Circular dsDNA	8.8	58.3	-	[2,26,68]
HRPV-6	*Hrr.* sp.	Persistent	Saltern, Thailand	Pleomorphic	48.5 ± 2.7	Circular ssDNA	8.5	62.7	-	[26,68]
SH1	*Har. hispanica*	Persistent	Salt lake, Australia	Spherical	70	Linear dsDNA	30.9	68.4	<5	[70,71,72,73,74]
HHIV-2	*Har. hispanica, Har. vallismortis, Har. japonica*	Virulent	Saltern, Italy	Spherical	80	Linear dsDNA	30.6	66.5	-	[2,75]
PH1	*Har. hispanica*	Persistent	Salt lake, Australia	Spherical	51	Linear dsDNA	28.1	67.6	-	[27]
SNJ1	*Natrinema* sp.	Temperate	*Nnm.* sp*.* (mitomycin C induction)	Spherical	67	Circular dsDNA	16.3	48.8-69.7	<6	[76,77]
His1	*Har. hispanica*	Persistent	Salt lake, Australia	Spindle	Head 44 × 77, Tail 7	Linear dsDNA	16.5	39-40	<7	[24,39,78]

Notes: ^1^ Viruses in bold have been studied in more detail, with both culture-dependent and culture-independent methods; ^2^ Strains previously designated as *Halobacterium halobium, Hbt. cutirubrum* and *Hbt. salinarium* were proposed to be corrected to *Hbt. salinarum* [79]; *Haloferax volcanii* was previously known as *Halobacterium volcanii*. Genus name abbreviations used are based on those proposed by the subcommittee on the taxonomy of the family Halobacteriaceae; the complete list: *Haladaptatus (Hap.), Halalkalicoccus (Hac.), Haloarcula (Har.), Halobacterium (Hbt.), Halobaculum (Hbl.), Halobiforma (Hbf.), Halococcus (Hcc.), Haloferax (Hfx.), Halogeometricum (Hgm.), Halomicrobium (Hmc.), Halopiger (Hpg.), Haloplanus (Hpn.), Haloquadratum (Hqr.), Halorhabdus (Hrd.), Halorubrum (Hrr.), Halosimplex (Hsx.), Halostagnicola (Hst.), Haloterrigena (Htg.), Halovivax (Hvx.), Natrialba (Nab.), Natrinema (Nnm.), Natronobacterium (Nbt.), Natronococcus (Ncc.), Natronolimnobius (Nln.), Natronomonas (Nmn.), Natronorubrum (Nrr.)* [80]; ^3^ The modes of infection listed here are tentative, with classifications of virulent infection possibly being persistent; ^4^ Proposed viral families were assigned based on morphology, with head-tailed (contractile), head-tailed (non-contractile), head-tailed (short), pleomorphic, spherical and spindle morphotypes assigned to *Myoviridae, Siphoviridae, Podoviridae, Pleolipoviridae, Sphaerolipoviridae* and *Fuselloviridae*, respectively. *Pleolipoviridae* and *Sphaerolipoviridae* families, and the classification of many haloarchaeoviruses into existing families, have not yet been formally accepted by the International Committee on Taxonomy of Viruses; “-”, not determined.

## 3. Morphotypes

### 3.1. Head-Tailed Caudoviruses

Head-tailed viruses (*Caudovirales*) comprise 96% of known bacteriophages and have been well studied compared to other viral morphotypes [3]. Head-tailed viruses are non-enveloped, and contain an icosahedral head which encapsidates the viral genome and is attached to a flexible hollow tail by a connector [81]. The head, known as the capsid, is composed of capsomer subunits, formed by main capsid proteins (MCPs).

Head-tailed viruses adsorb to cells by their tails and inject their DNA into the host. After transcription, translation and DNA replication, the virus capsid is assembled with scaffold proteins, DNA is packaged into the capsid, and the tail is added to the head. In some head-tailed viruses, the scaffold function is performed by a separate domain of the MCP or prohead protease, with this scaffold component removed from the capsid after assembly is completed [82,83,84]. The host cell is then lysed to release progeny viruses. Unlike head-tailed bacteriophages [81], mechanisms underpinning the life cycles of haloarchaeoviruses have not been well described. One major difference to the typical lytic cycle mechanism of bacteriophages is that some head-tailed haloarchaeoviruses are able to exit hosts without causing lysis (also see “Virulent and persistent infection” below).

Microscopic examination of environmental samples revealed that head-tailed viruses are the least abundant morphotype, comprising only 1% in some environments, yet the majority of isolated haloarchaeoviruses are head-tailed viruses [6,11,19,21,22]. This bias in part reflects the limitations imposed by difficulties in host cultivation, and virus isolation methods favouring lytic viruses that can be observed through the formation of plaques.

Head-tailed haloarchaeoviruses are morphologically and genetically similar to bacteriophage families, and are proposed to be assigned to the families *Myoviridae, Siphoviridae* and *Podoviridae*, which possess long contractile tails, long non-contractile tails and short tails, respectively [1,2,13,47,84,85]. Because archaeal caudoviruses carry many genes with homologues to bacterial genes, and the occurrence of head-tailed archaeoviruses is rare, it was suggested that caudoviruses did not evolve independently in *Bacteria* and *Archaea*, but instead were introduced to haloarchaea by interdomain spreading from halophilic bacterial hosts [13,22,84]. However, it has also been proposed that the presence of homologous genes in viruses from across the three domains of life is unlikely to be a consequence of inter-domain horizontal gene transfer due to the large evolutionary distances between cellular hosts [86]. Instead, homologous genes between archaeoviruses and bacteriophages have been regarded as derived from a primordial viral gene pool that predated the divergence of the three domains [86]. Head-tailed viruses (including distinct myoviruses and siphoviruses) could have been present at the very beginning of *Archaea* [87]. However, the evolution of head-tail viruses in haloarchaea might be complex and consist of vertical descent from a shared ancestor with bacterial caudoviruses along with subsequent inter-domain horizontal gene transfer, with the latter possibly occurring via recombination between intracellular archaeoviral genomes and provirus-containing exogenous bacterial DNA taken up by archaeal cells [87].

For the 17 head-tailed haloarchaeoviruses with sequenced genomes, a further subdivision has been made into groups including HF2-like myoviruses, HRTV-7-like myoviruses, HCTV-1-like siphoviruses, and “singletons” based on genome, structural and protein similarities [88]. Not all head-tail viruses have been classified into *Myoviridae, Siphoviridae* or *Podoviridae* because insufficient information is known about their tail structures (Table 1).

A total of 58 head-tailed haloarchaeoviruses have been described, although not all have been studied both morphologically and genomically. Head diameters range from 40 to 100 nm, and all three tail types have been characterized. With two recent culture-independent studies, the number of known haloarchaeoviruses had almost tripled [2,12]. A total of 42 viral genomes were retrieved by metagenomic sequencing using fosmid clones [12]. Of these, 17 genomes are almost complete and tentatively identified as head-tailed viruses due to the presence of terminase genes which are commonly used in caudoviruses for DNA packaging into capsids. Through culture-dependent methods, 26 new head-tail haloarchaeoviruses were identified from salterns and salt ponds in Italy, Thailand, Israel and Spain [2].

### 3.2. Pleolipoviruses

Recently, the seven pleomorphic haloarchaeoviruses were reviewed and proposed to be classified into a new group called pleolipoviruses (family *Pleolipoviridae*) due to their unique lipid envelope and protein similarities [26]. The members of this group are not restricted to *Archaea*; based on their structural features, this viral lineage is proposed to include the pleomorphic mycoplasma virus L172 [69,85,89,90]. However, there is currently no genome sequence data available for L172 to confirm this proposed relationship [85].

Pleolipoviruses have shapes ranging from elongated to round, and they possess a random distribution of spikes decorating their surfaces [26,69]. In contrast to most pleomorphic bacteriophages, pleolipoviruses contain no major structural proteins associated with their genomes, indicating that they have no nucleocapsids [26]. However, pleolipoviruses all contain two or three major structural proteins: at least one spike protein (usually one, although His2 has two), which appears to be important for host infection; and one or two membrane proteins, which may interact with the genome during virion assembly [26,85,89]. Post-translation modification of spike proteins has been shown for HGPV-1 and His2 (lipid modification) and HRPV-1 (glycosylation) [26,89].

Pleolipoviruses have a persistent mode of infection, and virions are released continuously from the host cell without lysis [2,26,69]. They are likely to be released from the cell by vesicle formation and budding, with nonselective acquisition of lipids from the host cell membrane [26,35,89]. In this respect, pleolipoviruses differ from the halosphaerovirus SH1, in which the lipid membrane underlies the icosahedral protein capsid (see “Lipids”, below) and lipids are acquired selectively from the host lipid pool, likely as a result of the geometrical constraints within the icosahedral protein capsid [71]. Described as “sloppy” assembly, this results in asymmetric virion structures, and varied genome types and genome packaging densities [26]. Accordingly, pleolipoviruses have a variety of genome types, including circular and linear, dsDNA and ssDNA [26,68].

Pleolipoviruses have been classified into two sets of subgroups, one based on virion structure dissociation, and the other based on genomic similarities (Table 2). These two subgroups do not fully correspond, indicating that the genetic material and replication strategies do not link to virion architecture. For the genomic set, the first two subgroups can form putative proviruses or proviral remnants (also see “Haloarchaeovirus genome variation” below).

**Table 2:** Pleolipovirus subgroups.

Subgroups Based on Protein Fragmentation After Virion Dissociation [26]	Subgroups Based on Genome Organization and Replication [68]
(1) Soluble fragments only	(1) Use rolling circle replication and contain the replication initiation protein (Rep)
HHPV-1, His2, HRPV-1	HHPV-1, HRPV-1, HRPV-2, HRPV-6
(2) Soluble fragments and one membrane-associated fragment	(2) Use rolling circle replication and do not contain Rep
HRPV-2, HRPV-6	HGPV-1, HRPV-3
(3) Membrane-associated fragments only	(3) Use protein-primed replication and have linear genomes
HGPV-1, HRPV-3	His2

### 3.3. Spherical/Icosahedral Halosphaeroviruses

Four spherical, tailless haloarchaeoviruses (family *Sphaerolipoviridae*) have been described; SH1, HHIV-2 and PH1 from *Har. hispanica* and SNJ1 from *Nnm.* sp. [27]. Two genera were proposed, separating SH1, PH1 and HHIV-2 into *Alphasphaerolipovirus*, and SNJ1 into *Betasphaerolipovirus* [91]. Halosphaeroviruses are structurally similar to the bacterial family *Tectiviridae*, suggesting that they may belong to the same evolutionary viral lineage [13,71,85]. However, SH1 has been proposed to represent a novel and early divergent lineage of viruses and virus-like genetic elements infecting halophilic archaea and thermophilic bacteria [42]. Recent evidence suggests that bacteriophages infecting thermophilic *Thermus thermophilus*, P23-77 and IN93, have similar morphologies, protein structure and conserved core genes, and should be included within the same family under the genus *Gammasphaerolipovirus* [91].

The viral particles range from 51 to 80 nm in diameter, and contain a capsid with an internal lipid membrane. SH1 has spike structures on the capsid, and putative spike proteins appear to be present in HHIV-2 and PH1 [27,43,71,73,75,77]. SH1, HHIV-2 and PH1 contain linear dsDNA with terminal proteins [27]. In contrast, SNJ1 contains circular dsDNA [77].

### 3.4. Spindle-Shaped/Lemon-Shaped Salterproviruses

The His1 virus is the only spindle-shaped haloarchaeovirus isolated to date, despite this morphotype being the most abundant in several hypersaline environments [6,19]. His2 was originally classed as spindle-shaped based on morphological similarities to His1. However, His2 has a different protein profile, and no gene synteny with His1, and was later identified to be pleomorphic, belonging to the pleolipoviruses [26,39].

The spindle-shape morphotype is unique to archaeoviruses and has not been described in bacteriophages or eucaryoviruses [78,92]. His1 has similar morphology to the archaeovirus family *Fuselloviridae*. However, genomic differences has established His1 as the type species of the new genus *Salterprovirus* [24]. Recently it has been proposed that all spindle-shaped viruses can be segregated into the families *Fuselloviridae* and *Bicaudaviridae*, and based on related capsid proteins and overlapping gene content, salterprovirus His1 should also be included within the *Fuselloviridae* family [92].

Flexibility is a common feature of spindle-shaped viruses, and in His1 this is due to lipid modification of the virion capsid proteins [39,92]. His1 was also observed to undergo structural transformation to form elongated particles [39,78,92]. These larger particles may contain more than one copy of the genome, or a less densely packaged genome [39]. Electron micrographs indicate the presence of a very short tail on His1 particles, although His1 is referred to both as short-tailed and tailless depending on the publication source [24,39,78,92].

Flexibility of virions has also been observed for spindle-shaped viruses infecting hyperthermophilic *Archaea*. *Acidianus* two-tailed virus (ATV), the only member of the family *Bicaudaviridae*, was shown to develop long tails at each pointed end of the spindle-shaped particle, after being released from the host cell [93]. The mechanism is driven by conformational changes and rearrangements of proteins within the virus particle [94]. The large spindle-shaped viruses of *Sulfolobus tengchongensis*, spindle-shaped virus 1 and 2 (STSV1 and STSV2), exhibit a single tail, with a “nose-like” structure at the opposite end of the spindle. The length of the tails of different virions varies between 20 and 500 nm [95,96], and the range of sizes may reflect the mechanism of virion release which may be via a budding mechanism involving host lipids [96]. The morphological similarities between the spindle-shaped archaeoviruses from hyperthermophiles and haloarchaeoviruses illustrate there is a great deal to be learned about the evolutionary relationships of this virus morphotype.

## 4. Macromolecules

### 4.1. Genomes

The majority of viruses listed in Table 1 have had their genomes sequenced, and a quarter of currently known haloarchaeoviruses were discovered by genome reconstruction of metagenome data from hypersaline environments [12]. In recent studies, 140 viral genomes were reconstructed from Lake Tyrrell (Australia) and were determined to have partial sequence similarities to previously sequenced haloarchaeoviruses BJ1, EHP-1 and φCh1 [97,98]. However, it was undetermined whether these sequences belonged to archaeoviruses or bacteriophages, and therefore they have not been discussed in this review. The sequenced haloarchaeoviruses all contain DNA, and genome size ranges from 7.0 to 103.2 kb (HCTV-1) [44,69]. The majority of genomes sequenced are linear, and all are dsDNA, except for HRPV-1 and HRPV-6 which are circular ssDNA [26,69].

Most open reading frames (ORFs) of haloarchaeoviruses lack significant matches to sequences in databases; for example, HF1 has 102 unique ORFs out of a total of 117 [25]. For genes with annotated functions, associations were made to organisms from all three domains of life, as well as viruses infecting all three domains of life, indicating that that haloarchaeoviruses have been involved in the exchange of genetic material that has originated from a very broad range of taxa [95]. One apparent consequence of this transfer and recombination is that many haloarchaeoviruses have mosaic genomes [47,50,99,100].

Because viruses use host replication machinery, it may be expected that their G + C mol% would be similar. In sequenced haloarchaeoviruses, the G + C mol% mostly ranges from 55% to 70%, which is similar to the host haloarchaea (60% to 70%) [12]. The G + C mol% of *Hqr. walsbyi* is lower than other haloarchaea, at 47.9%. Viral genome reconstruction from metagenome data identified 14 new haloarchaeoviruses with a relatively low G + C mol% of 43% to 46%, therefore possibly indicating they infect *Hqr. walsbyi* [12,101]. They may also infect members of a new order of haloarchaea which have low G + C mol% and were identified recently using metagenomics [102,103] and single cell sorting [104].

However, the G + C mol% of haloarchaeoviruses does not always reflect their hosts, and this may impact translation efficiency via changes in codon usage. HF2 has 10% lower G + C mol% than its hosts *Hrr. coriense* and *Hrr. saccharovorum*, but contains five putative tRNA sequences, which are thought to compensate for differences in codon usage [99]. Similarly, putative tRNA for phenylalanine is encoded in BJ1, and tRNA for glutamine in HSTV-2 and HVTV-1 [50,63]. In contrast, while His1 and His2 have G + C mol% ~ 20% lower than their host *Har. hispanica*, they do not appear to encode tRNAs [24]. This may indicate that His1 and His2 infect other (unknown) hosts with a lower G + C mol%. Interestingly, HGTV-1 encodes 36 tRNAs, covering all universal genetic code amino acids, but the significance of this is yet to be determined as the G + C mol% for its host (*Halogranum* sp. SS5–1) has not been established [47].

Matching their haloarchaeal hosts, the majority of haloarchaeovirus proteins predicted from genome sequences have predicted isoelectric points (p*I*) that are acidic (below pH 6). Haloarchaea accumulate high concentrations of intracellular salt (KCl). The low p*I* of proteins results in a negative charge which is thought to facilitate salt bridge formation and attract water molecules thereby forming hydration shells around proteins [105]. This property would enable haloarchaeovirus proteins to be compatible with both the high intracellular and extracellular salt environments [106]. The low p*I* combined with covalent cross-linking of structural components may promote protein stability more broadly, enabling haloarchaeoviruses to cope with environmental changes, such as transient low concentrations of salt [52] (also see “Salinity, infection and evolutionary strategy” below). As the extremely halophilic bacterium, *Salinibacter ruber* has protein p*I* values, DNA G + C mol%, and intracellular KCl concentrations similar to haloarchaea [107], the bacteriophages that infect *S. ruber* may have similar stability properties to haloarchaeoviruses.

### 4.2. Lipids

From the limited analyses of lipids in haloarchaeoviruses it has been determined that caudoviruses typically do not contain lipid membranes, pleolipoviruses contain an outer lipid envelope, halosphaeroviruses contain a lipid membrane inside the protein capsid, and salterprovirus His1 and some halosphaeroviruses contain lipid-modified proteins [108]. The presence of an outer lipid envelope, such as in pleolipoviruses, allows virus particles to have mechanical flexibility, thereby enabling exit from host cells without causing lysis [26] (also see “Spindle-shaped/lemon-shaped salterproviruses” above). The His1 salterprovirus does not contain a lipid bilayer, but the lipid-modified capsid protein also appears to enable virion flexibility and persistent infection of host cells [39] (also see “Virulent and persistent infection” below).

Viral lipids are generally acquired from the host lipid pool. Pleolipoviruses show nonselective incorporation of host lipids, and appear to be released from the host cell by budding [26,35,89]. In contrast, halosphaeroviruses selectively acquire lipid components from the host cell membrane [71,77]. The halosphaerovirus SH1 contains only negatively charged phospholipids as an inner lipid membrane beneath the capsid, which may help to neutralize and stabilize the membrane, thereby facilitating membrane-DNA interactions [43].

### 4.3. Structural Proteins of Head-Tailed Haloarchaeoviruses

The majority of known haloarchaeoviruses have head-tail morphology, similar in structure to head-tailed bacteriophages (*Caudovirales*) [1,13]. The virus particle/virion consists of the head (capsid) which houses the genetic material of the virus, and a tail which is responsible for host attachment.

#### 4.3.1. Capsid Proteins

Several novel capsid structures have been observed in haloarchaeoviruses. In order to expand the capsid size to accommodate more genetic material, three main mechanisms are employed by bacteriophages; increasing the number of protomers, *i.e.*, increasing the triangulation number (T-number); increasing the size of each MCP; or assuming an elongated, prolate structure rather than icosahedral [50]. In HSTV-2, a fourth solution was discovered: using minor capsid proteins to form trimeric structures in between protomers to expand the construct of the capsid [50]. Hence the genome size of HSTV-2 is much larger than the expected size for a capsid structure of T = 7. Similar capsid constructs possibly also occur in HF1 and HF2 [50].

Some haloarchaeovirus capsids contain additional proteins. HVTV-1 contains decorative trimeric structures in the center of each MCP hexamer to increase capsid stability [50]. HSTV-1 has large, cone-shaped tower structures extending from the surface, but the function of these is not known [38]. Some halosphaeroviruses such as SNJ1 and SH1 have two major capsid proteins [71,77]. SH1 was also found to have an unusual capsid architecture of T = 28 with horn-like spikes which decorate and stabilize capsomers that constitute the capsid [43]. The capsid of SH1 is composed of proteins with single vertical β-barrel folds, and this structure provides a possible “missing link” in the evolution to double β-barrel folds which are commonly present in viruses infecting all three domains of life [43].

#### 4.3.2. Tail Proteins

The tail length of bacteriophage λ is determined by the tail tape measure protein [109]. Putative tail tape measure proteins were identified in the genome sequences of ten head-tailed haloarchaeoviruses, suggesting a common mechanism for determining tail lengths [47]. The caudoviruses HF1 and HF2, which are nearly identical in the first 48 kb of their genome, have many changes in the last 28 kb [25]. The structural proteins for virion assembly appear to be highly conserved whereas other proteins such as tail fibers are highly variable [25]. This offers an explanation for the different host ranges of these two viruses despite being so similar morphologically and genetically.

**Figure 2:**
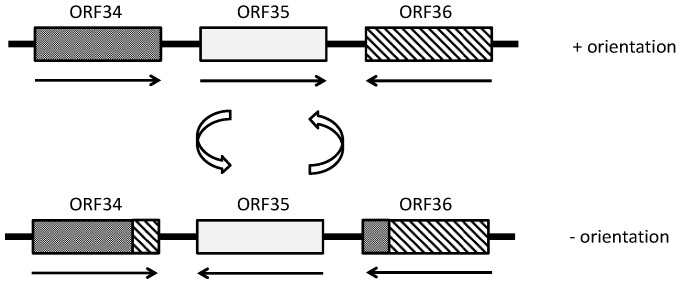
Schematic representation of the inversion of ORF35 and its effects on ORF34 and ORF36 in ΦCh1. ORF34 and ORF36 code for tail fiber proteins and ORF35 is invertible and codes for a recombinase. The black arrows indicate the orientation of the ORFs. The white arrows symbolize an inversion of ORF35, after which the ends of ORF34 and ORF36 are swapped, causing variations in resulting tail fiber proteins. Adapted from [100].

ΦCh1 exhibits phase variation in tail fiber proteins, thereby enabling constant changes to be manifested in tails produced [53]. This may overcome host defenses to improve attachment and infection [53] (also see “Host defense” below). Tail fiber proteins in ΦCh1 are encoded by the genes ORF34 and ORF36, which are separated by ORF35, and are oriented with their 3’ ends facing ORF35 (Figure 2). ORF35 codes for recombinase and is invertible, causing variations in the 3’ ends of ORF34 and ORF36. The resulting tail fiber proteins have variable lengths and C-terminal regions [100]. ΦCh1 that is able to infect *Nab. magadii* only has ORF35 in the negative orientation, and ORF34 expressed. The tail fiber proteins commonly contain a galactose-binding domain in the C-terminal region. This may facilitate attachment to the host as galactose residues are present in the S-layer and flagella of *Nab. magadii*.

## 5. Life Cycles

### 5.1. Adsorption and DNA Ejection

Spikes which are present on the surfaces of pleolipoviruses and some halosphaeroviruses, have been characterized in detail in HRPV-1 and SH1, respectively, and their function has been linked to infectivity and host adsorption [26,43,89]. Haloarchaeoviruses studied have adsorption rates ranging from 1.2 × 10^−10^ to 2.9 × 10^−13^ mL/min, which is low compared to 10^−9^ mL/min for bacteriophage λ [48,64,110]. It was suggested that such low adsorption rates may be due to differences in surface structures of *Bacteria* and *Archaea* making it more difficult for haloarchaeoviruses to attach [48]. The low adsorption rates may reflect an evolutionary adaptation to aspects of host growth dynamics; in particular, long generation times which may deplete total host numbers if rapid adsorption (and hence infection) rates occurred [48]. Alternatively, it has been hypothesized that the changing salinity conditions of some hypersaline environments may have selected for slow-adsorbing viruses that can adsorb effectively over a range of salt concentrations [111] (also see “Salinity, infection and evolutionary strategy” below).

Very limited information is available on archaeovirus DNA ejection, and the first in depth study was performed in 2013 on His1 [112]. His1 DNA ejection into the host is dependent on osmotic pressure, and the ejection rate is reduced with increased external pressure [112]. Due to the stabilizing effect of charge screening, increases in external positive ion concentration decreases the number of ejections and the length of ejected DNA [112]. Additionally, it was suggested that cellular processes are required to complete the DNA transfer, as *in vitro* ejections were found to not be complete [112]. DNA ejection was directional, and occurred at a mean rate of 144 ± 72 kbp/s in His1 compared to 60 kbp/s in bacteriophage λ [112,113]. The ejection of DNA was stepwise, and seemingly random stops and starts in the process were attributed to conformational changes or deformations of the capsid [112].

### 5.2. Replication and Assembly

The mechanisms of replication and assembly of haloarchaeoviruses have mainly been inferred from genetic analyses. Both rolling-circle replication (HHPV-1 and HRPV-1) and protein-primed replication (His2, SH1, HHIV-2, ΦN and PH1) have been proposed [24,27,35,66,75]. The genomes of HF2, HSTV-2, HCTV-1, HCTV-5, HRTV-5, HRTV-7 and HRTV-8 contain terminal repeats, suggesting replication occurs through concatameric intermediates [46,47,50]. In contrast, the closely related ΦH, ΦN and ΦCh1 viruses, and HCTV-2, HHTV-1, HHTV-2, HRTV-4 and HSTV-1 have partially circularly permuted, terminally redundant genomes indicating they are likely to be packed by a headful mechanism [38,47,54,66].

A number of haloarchaeoviruses, including His2, SH1, HHIV-2, ΦN and PH1, contain proteins covalently-bound to the termini of their linear genomes that may be involved in protein-primed replication [24,27,66,75]. His2 also encodes genes with similarity to DNA polymerases from viruses and plasmids known to replicate via protein priming, thereby providing support for this hypothesis [24]. Interestingly, the 5’ termini of His2 and SH1 genomes must be protein bound in order for the viral DNA to be successfully artificially introduced into host cells [70]. However, terminal proteins might function by end-patching genomic DNA after replication, rather than in primary replication [27].

The members of pleolipoviruses and members of halosphaeroviruses have different genome forms and methods of replication [26,27]. For example, the pleolipoviruses HHPV-1 and HRPV-1 have ssDNA and dsDNA, respectively, but they share sequence homology and synteny for genes related to host integration, replication and virus assembly functions [35]. Because these viruses acquire host cell membrane lipids and are released from the host by budding, the form of the genome does not appear to be important to the assembly process [26].

### 5.3. Temperate Infection

Only three temperate haloarchaeoviruses have so far been discovered: ΦCh1, SNJ1 and ΦH [51,76,110]. HCTV-5, HRTV-5, HRTV-7 and HRTV-8 encode putative integrases, but have not been found to form lysogens [47]. Based on the turbidity of its plaques and the presence of putative integrase genes in the genome, BJ1 may be a lysogen, but further study is required to assess this [63]. The lack of isolation of temperate haloarchaeoviruses is consistent with metagenomic analyses of a seawater evaporation pond in the Santa Pola saltern (Spain) which identified the presence of integrases in only 2 out of 42 viral fosmids [12].

ΦCh1 integrates into the genome of *Nab. magadii*, while SNJ1 in *Nnm.* sp. and ΦH in *Hbt. salinarum* exist as a lysogen in a non-integrated circular form [51,76,114]. ΦCh1 contains a putative site-specific λ-like integrase which probably facilitates integration [52]. Non-integrated, circular forms of viruses/phage are often described as plasmids because they are semi-autonomous, extrachromosomal DNA that replicate in a similar fashion to plasmids, such as *Escherichia coli* phage P1 [115].

The circular form of SNJ1 (referred to as plasmid pHH205) confers to its host resistance to superinfection [76,77]. ΦH circularizes either as a full-length form, or just the L segment (referred to plasmid pΦHL) [114] (also see “Gene regulation” below). Although ΦH does not integrate into the host chromosome, recombination with other plasmids containing homologous segments can occur, and ΦH variants with insertions, deletions and inversions have been identified [54,116].

Two putative head-tailed proviruses, Hlac-Pro1 and Nmag-Pro1, were identified in the genomes of *Hrr. lacusprofundi* and *Nab. magadii*, respectively [84]. Both putative proviruses share sequence similarities with head-tailed haloarchaeovirus BJ1, which infects *Hrr.* sp., and Hlac-Pro1 appears to be defective [63,84]. Viral sequences matching to BJ1 were also detected in the metagenome of Deep Lake (Antarctica), the hypersaline system where *Hrr. lacusprofundi* was isolated from and represents about 10% of the cellular community [117].

Putative proviruses in haloarchaea relating to sphaerolipoviruses have been described. HalaPauP1 from *Haladaptatus paucihalophilus* and HaloLacP1 from *Halobiforma lacisalsi* have homologous genes to alphasphaerolipoviruses, while HaloMukP1 and HaloMukP2 from *Halomicrobium mukohataei*, and integrated *Haloarcula* provirus (IHP) from *Haloarcula marismortui* shares genes with betasphaerolipovirus SNJ1 [91].

Putative pleomorphic proviruses were also found as extrachromosomal genetic elements in *Hfx. volcanii* and *Hrr.* spp. [2,118,119]. The sequence of plasmid pHK2 from *Hfx. volcanii* and a chromosomal region of *Hfx. volcanii* have considerable identity to pleomorphic viruses HHPV-1 and HRPV-1. The chromosomal region of *Hfx. volcanii* and pHK2 contain putative integrases, suggesting that it is a virus that can both integrate and replicate extrachromosomally [35]. Similarly, plasmid pHRDV1 from *Hrr.* sp. has a genome organization and protein homology linking it to pleomorphic viruses HRPV-3 and HGPV-1 [119]. Although pHK2 and pHRDV1 have not been shown experimentally to form extracellular viruses, these findings suggest that temperate haloarchaeoviruses may be more prevalent than is currently recognized.

### 5.4. Virulent and Persistent Infection

The majority of head-tailed haloarchaeoviruses are described as virulent (see Table 1), but the label may be inappropriate for many, since most haloarchaeoviruses do not portray the normal characteristics expected of virulent viruses (also see “CLASSIFICATION” above). In pleolipoviruses, salterprovirus His1 and some halosphaeroviruses and head-tailed haloarchaeoviruses, progeny can be produced continuously without lysing host cells, and are described as causing persistent (or chronic) infections [111]. Those presently characterized as persistent include head-tailed HF1, HF2, Hs1, Hh-1, Hh-3 and S5100, all known pleolipoviruses, halosphaerovirus SH1 and PH1, and salterprovirus His1 (Table 1).

By using a budding mechanism, pleolipoviruses are released to the extracellular environment while the host continues to grow [69,89]. As haloarchaea lack a rigid exterior, such as the peptidoglycan layer present in many bacterial cell walls, haloarchaeoviruses with protein capsids may enter and exit haloarchaea without disrupting the integrity of the cell envelope [64]. For His1, His2 and SH1, virus particles are continually liberated from host cells without cell lysis, but cells eventually die and lyse [24,72,111]. During persistent infections, host cells continue to multiply at a slightly slower growth rate than when non-infected, and cells are not immune to superinfection by heterogeneous viruses, eventually lysing when the cell membrane is compromised by continual entry and egress of viruses [24,25,64,111]. Regulation of transitions from persistent to lytic states is not well understood, but for His1 and S5100, salinity has been shown to be a controlling environmental factor [49,62] (also see “Salinity, infection and evolutionary strategy” below). For lytic haloarchaeal caudoviruses, the precise mechanism causing host lysis is unclear, as they lack known lysin or holin genes (as described for many bacteriophages). RTX toxins, which are inferred to be cytolytic, have been annotated in the genomes of certain haloarchaea (e.g., a putative *Hqr. walsbyi* virus [12]). Genes for zeta toxins have been identified in a number of head-tailed haloarchaeoviruses (e.g., HVTV-1, HCTV-1), but their role in the life cycle of the virus is not known [50]. It has not been determined if haloarchaeovirus encoded RTX or zeta toxins play a role in host cell lysis.

## 6. Genetic Manipulation and Gene Expression

### 6.1. Genetic Manipulation

A method to artificially introduce ΦH DNA into *Hbt. salinarum* was developed using polyethylene glycol and spheroplasts [120]. This was extended to *Hfx. volcanii* which was otherwise unable to be infected by ΦH particles [121]. The DNA of several other haloarchaeoviruses, including His2 and SH1, has also been successfully introduced into hosts [70]. The successful introduction of viral DNA by artificial means, and subsequent generation of virus progeny, highlights the relatively broad range of hosts that are capable of sustaining haloarchaeovirus replication.

The broad host-range ability has been recognized for its potential to facilitate the development of ΦH-based shuttle vectors for haloarchaea [70]. Using the replicon region of ΦH, plasmid vectors were developed for transforming *Hbt. salinarum*, enabling studies of gene regulation via experiments using transposon mutagenesis [122]. However, in *Hfx. volcanii*, while the recombinant plasmids were able to replicate they tended to be lost from the host. The outcomes of these studies to date provide a strong basis for further development of genetic manipulation systems for haloarchaea.

### 6.2. Gene Regulation

To date, in-depth molecular studies of gene regulation in haloarchaeoviruses have only been performed on ΦH and ΦCh1, revealing complex mechanisms of gene regulation. In *Hbt. salinarum*, ΦH appears to have evolved mechanisms to regulate host abundance and protect itself during unfavorable growth conditions (e.g., changes in salinity) [57]. ΦH is temperate and exists either as a 57 kb circular, extrachromosomal provirus, or as a 12 kb L circular form which only comprises the L region of the ΦH genome. The presence of either confers host immunity against lysis by ΦH.

**Figure 3:**
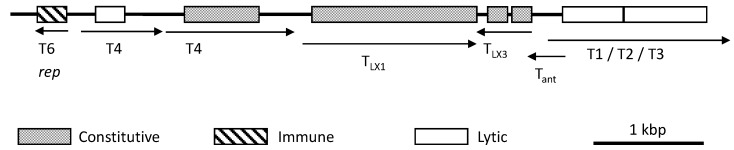
Schematic map of the genetic region between transcripts T6 and T1 in ΦH. Boxes represent ORFs. Adapted from [59].

The L region encodes the majority of viral regulatory functions, and carries genes responsible for influencing whether lytic or lysogenic cycles occur [59] (Figure 3). In the L region, the T4 gene is required for lytic growth, and is located back-to-back with the repressor *rep* gene [57]. Expression of T4 is very effectively turned off by the product of the *rep* gene, which binds to the T4 promoter region. T4 and *rep* transcription seem to be mutually exclusive, as *rep* transcription is also repressed by T4 transcription [61]; a phenomenon reminiscent of CI and Cro proteins in *E. coli* λ phage. Expression of T4 would maintain lysogeny of ΦH and cause immunity by preventing infecting ΦH-like viruses from expressing genes necessary for entering the lytic cycle. The *per* (promotes efficiency of *rep*) gene enhances the effect of the Rep protein tenfold [59]. However, the host immunity conferred by the L form can be overcome by some ΦH mutants, such as ΦHL, which have an ISH23 inserted element between the T4 and *rep* genes [57]. This separates the repression binding site (operator region) from the promoter region of T4, thereby rendering Rep activity null [57].

An additional mechanism for immunity in hosts is through post-transcriptional processing by host endonucleases of the transcript from the early lytic gene, T1 [59] (Figure 3). Antisense RNA is transcribed from the T_ant_ gene located at the end of the T1 gene, expressed from the complementary strand in the opposite direction to T1 [58]. Binding of the antisense RNA makes T1 susceptible to specific host RNAses which cleave ribosomal binding sites from the first ORF, but interestingly does not digest the products [58].

In the temperate virus ΦCh1, ORF49 is regulated by the *rep* gene, which encodes the winged-helix repressor Rep protein [123]. Proteins Gp43 and Gp44 encoded by ORF43 and ORF44, respectively, partially relieve *rep*-mediated repression as they bind to the same site as the Rep protein, but at a lower specificity.

While gene regulation studies are limited, the parallels to control mechanisms in head-tail bacteriophages, such as *E. coli* λ, are notable. Such mechanisms have not been discovered in viruses infecting thermophilic archaea, which may indicate that head-tail haloarchaeoviruses have more in common with bacteriophages.

## 7. Host-Virus Responses

### 7.1. Host Defense

In the ongoing struggle between viruses and hosts, hosts develop mechanisms to defend against viruses while viruses evolve to evade host defenses. One well studied mechanism used by bacterial hosts against bacteriophages is restriction-modification systems [124]. Many haloarchaeoviruses have a lack of, or low incidence of, the sequence GATC and its inverse CTAG, which may relate to the tetranucleotide sequences being commonly targeted by restriction endonucleases [124]. This phenomenon occurs in all groups of haloarchaeoviruses, including caudoviruses BJ1, HF1, HF2, pleolipovirus HHIV-2, halosphaerovirus His2, PH1 and SH1, and salterprovirus His1 [24,27,46,63,99]. Furthermore, HF2 possesses AT rich sequences which may also help to circumvent host defense mechanisms as haloarchaeal restriction systems mainly target G + C mol% rich regions [46]. Additionally, some haloarchaeoviruses have been observed to carry modified bases, including methylated cytosines in ΦN and HRPV-3 and methylated adenine in ΦCh1 and HGPV-1, which may reduce susceptibility of their DNA to host endonucleases during infection [51,66,68]; methylation could arise from host- or virus-encoded methylases. However, the motif GATC is 50% G + C mol%, and the majority of haloarchaea have >65% G + C mol% with the third codon position reaching >90%, while *Hqr. walsbyi*, has G + C mol% < 50% with the third codon position reaching down to 42%. The observation that haloarchaeoviruses do not tend to contain the sequence GATC may therefore relate to their biased G + C mol% content.

Genomic and metagenomic data have provided evidence that hosts evade haloarchaeovirus invasion by expressing cell envelope proteins which have sequence variation. This has been observed for *Hqr. walsbyi* [125] and within haloarchaea communities [12], and similar observations were made for the bacterium *S. ruber*, based on metagenome data from seawater evaporation ponds of Chula Vista salterns (USA) [107]. In *Har. marismortui*, the subunit (archaellin) composition of the flagellum (archaellum) is known to vary, possibly as a mechanism to facilitate archaellin switching in order to evade viruses that target archaella [126].

**Figure 4:**
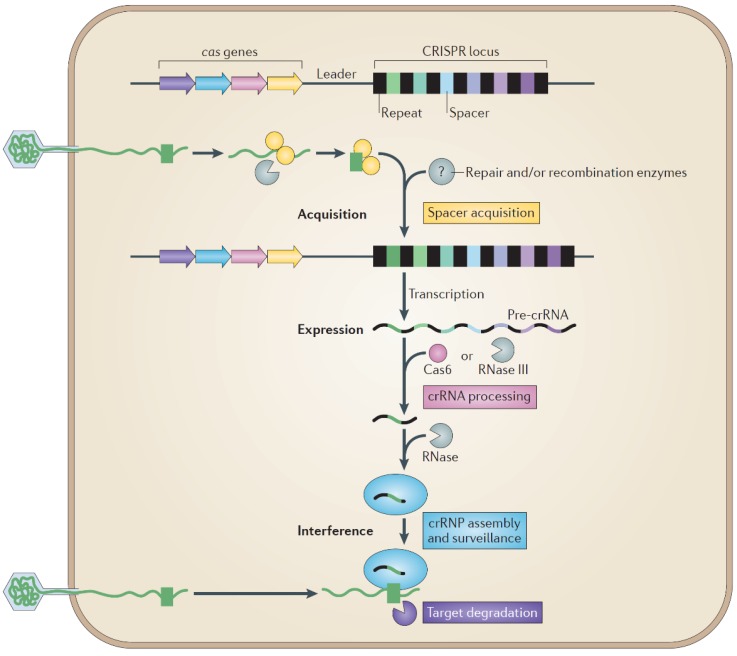
Schematic of the CRISPR-Cas adaptive immune system [131].

Clustered regularly interspaced short palindromic repeats (CRISPR) and CRISPR-associated (Cas) proteins form a CRISPR/Cas system that functions as a defense mechanism against invading foreign nucleic acid [127,128] (Figure 4). CRISPR/Cas systems have been reported in about 80% of *Archaea* and 50% of *Bacteria* examined [127,128], and have similarities to the RNA interference systems of *Eucarya* [129]. The CRISPR/Cas systems differ to other defense systems of *Archaea* and *Bacteria* by being dynamic and responsive to new invading DNA, and being sequence specific [130].

Detailed CRISPR/Cas reviews have been published [131,132,133,134,135], some with more emphasis on archaeal systems [136,137,138]. Briefly, the system consists of three stages; adaptation (or acquisition), expression and interference, which involve different Cas proteins (Figure 4). In the adaptation stage, small fragments of foreign nucleic acids termed protospacers are recognized as non-self, and the corresponding sequences incorporated into a host genome CRISPR locus as spacers in between short direct repeat sequences. Short sequences called protospacer adjacent motifs (PAMs), directly upstream of protospacers, are essential for the selection of new sequences to be incorporated as spacers. In the expression stage, the CRISPR locus is expressed to generate pre-CRISPR RNAs (pre-crRNAs), which are processed into mature crRNAs. In the interference stage, crRNAs are used to recognize invasion by the same foreign nucleic acid elements for subsequent destruction or silencing.

Type I and type II CRISPR/Cas systems have been identified in *Archaea*, with type I-B mainly observed in haloarchaea (most studies have been performed on *Hfx.* spp.) [127]. *Hfx. volcanii* strain H119 contains a type I-B CRISPR/Cas system with eight Cas proteins and three CRISPR loci with a total of 74 spacers, which are constitutively transcribed and processed [130,139]. Similar CRISPR repeat sequences were found in 20 of 32 haloarchaeal genomes [130,139,140], and CRISPR/Cas systems were identified in about two thirds of sequenced haloarchaeal genomes [127,128]. In *Haloferax mediterranei*, six CRISPR loci were identified, and the CRISPR/Cas system components and mechanisms were different from other type I-B systems in methanogens and *Clostridium* spp., suggesting that haloarchaea may have unique features in their CRISPR/Cas systems [141].

In contrast to the multiple loci in *Hfx. mediterranei*, a recent study of *Hrr.* spp. phylogroups reported that the distribution of CRISPRs was variable within phylogroup A and the genus as a whole, and phylogroup B members appeared to lack CRISPRs entirely [142].

In studies identifying CRISPR/Cas systems in haloarchaeal genomes, only few haloarchaea spacers matched to haloarchaeovirus sequences [127,128]. In a metagenomic study of Lake Tyrrell (Australia), ~ 8000 unique spacer sequences were identified, but the vast majority had no matches to viral contigs from the same sample, and the spacers with matches to viruses did not match sequences in archaeal genomes from the same environmental sample [98]. In a separate study of ten haloarchaeovirus genomes, no matches were identified to CRISPR spacers in haloarchaeal genomes [47].

The low level of match between spacers (haloarchaea genome sequences) and protospacers (haloarchaeovirus sequences) may be due to CRISPR/Cas systems successfully maintaining viral populations at low abundances, therefore rendering most of the invading viruses undetectable in metavirome data [98]. It is also possible that only few haloarchaeovirus sequences are available, and viruses have fast rates of evolution, and therefore protospacer-spacer matches may not be recognized in database searches [130,139,140].

However, the CRISPR/Cas systems may not be the primary resistance mechanism against viruses in haloarchaea [47,142]. Some CRISPR systems require precise matches between spacer and invading proto-spacer [143], and the cost of maintaining the system may outweigh the benefit [142]. Moreover, haloarchaea are able to form heterodiploids and exchange long tracts of DNA [144], and exchange has been observed in environmental communities even between distinct haloarchaea genera [117]. As CRISPR/Cas systems function by degrading incoming DNA, if DNA exchange benefits the community (and is therefore positively selected), it is unclear what role CRISPR/Cas systems would perform and how they would be regulated in response to self, similar or dissimilar “invading” DNA. Overall, the defense systems observed in haloarchaea may reflect a dynamic balance between CRISPRs and other defense systems (e.g., restriction/modification systems, cell surface variation) and the selection pressures related to the nature and functionality of those systems in response to the cost/benefit of the invading DNA.

### 7.2. Host-Range

Assessments of host-range are limited by the ability to cultivate hosts and successfully infect and propagate haloarchaeoviruses using available hosts. Moreover, if plaque formation is used to assay infection, persistent or temperate infection is difficult to assess and therefore interpret host-range. Most haloarchaeoviruses have been reported to have a narrow host range, and this has been attributed to the small receptor range of virus attachment proteins rather than due to DNA restriction barriers [45]. However, in a study of infectivity, host-range and biogeography, myoviruses were found to be most readily isolated on a *Hrr.* sp. host, yet cross-testing revealed the ability of some of the isolated myoviruses to infect *Har.* sp., *Halosarcina* sp., *Hgm.* sp., *Halogranum* sp. and two unclassified isolates [2,12]. While host-range varied with virus type (e.g., myoviruses had a wider host range than siphoviruses), the study highlighted that, given the opportunity, haloarchaeovirus-mediated exchange of host and viral material is likely to be able to occur between diverse members of communities that are from distinct geographic locations [2,12].

### 7.3. Haloarchaeovirus Genome Variation

As an extension to the concept of ecotypes and niche adaptation which allows a population to adapt to differences or changes in environmental parameters, the term “ecoviriotypes” was proposed to describe viruses which have different but very closely related genomes (e.g., single nucleotide polymorphisms-SNPs) [141]. Metagenomic and metatranscriptomic analyses of haloarchaeoviruses in a seawater evaporation pond in a multi-pond solar saltern in Santa Pola (Spain) determined that the mutation frequency was 7.7 × 10^−3^ substitutions per nucleotide, with 24% higher mutation frequency in coding regions than non-coding regions [101,145]. As mutation rates of DNA bacteriophages were reported to be between 10^−8^ to 10^−6^ substitutions per nucleotide [146], this indicates that haloarchaeoviruses can have abnormally high mutation rates and those populations require high adaptive potential [101]. Such high rates of mutation may contribute to the generation of phenotypic variation in haloarchaeoviruses (e.g., tail proteins), with variant forms selected in response to host evasion mechanisms, such as hosts expressing variant forms of cell envelope proteins (see “Host defense” above).

The term virus and plasmid related elements (ViPREs) was coined to describe virus or plasmid integrants in the host genome which form gene cassettes that can move between viruses during recombination, thus allowing novel virus genomes to be generated by re-assortment [27]. Such recombination events have been proposed to explain the fact that viruses in closely related groups (within halosphaeroviruses or pleolipoviruses) often have similar capsid proteins but different genomic characteristics and methods of replication [26,27,147]. The first ViPREs were described in *Hqr. walsbyi* and subsequently described in *Hmc. mukohataei* and *Hfx.* spp. [27]*.* These findings indicate that ViPREs are involved in recombination not only between virus species, but also across host species. By enabling extensive recombination between viruses, plasmids and hosts, ViPREs represent mobile collections of capsid and replication genes from different sources, which may allow rapid evolution of haloarchaeoviruses and hosts.

### 7.4. Salinity, Infection and Evolutionary Strategy

Due to their proportionally high numbers, viruses have the potential to provide strong selective pressures on hosts [28]. To some extent this may be counterbalanced by haloarchaeoviruses tending to adopt persistent, rather than lytic, infection of hosts (also see “Virulent and persistent infection” above). While virus exploitation of their hosts may drive the selection of defense mechanisms, viruses may benefit host populations by acting as agents of genetic exchange and therefore influencing populations in positive ways [19,69,148]. As a measure of viral impact, one study of the microbial loop demonstrated that production by *Archaea* and *Bacteria* exceeded losses caused by viral lysis [6]. Viral concentrations have also been linked to growth responses of hosts in response to environmental conditions, such as levels of salinity [11,19,48].

As freshwater input can cause major changes to salinity in hypersaline environments, numerous studies have examined the effects of salinity on virus-host interactions. Membrane-containing viruses were found to be more sensitive to low ionic strength environments compared to head-tailed viruses [48]. In particular, enveloped pleolipoviruses were more sensitive than halosphaeroviruses with internal membranes [48]. Nevertheless, all haloarchaeoviruses have been shown to be able to withstand wider variations in salinity than their hosts [5,48,64]. In fact, His1 can maintain a constant titer for 24 h at 0.02% salinity, and HHIV-2 can maintain infectivity in as low as 0.006% NaCl [64,78]. Furthermore, HHTV-1, HCTV-1, HRTV-1, ΦN and SH1 are reported to maintain 50% infectivity in distilled water [48,66]. The ability to endure exposure to low salinity allows haloarchaeoviruses to survive influxes of freshwater that would be lethal to hosts, offering a possible evolutionary advantage to the viruses [64].

The infectivity of HVTV-1 and HSTV-2 is salt-dependent and low salinity causes reversible inactivation of the virus [50]. As a result, the level of active viruses varies with the numbers of viable hosts and the chance of host extinction is reduced. These findings are corroborated by studies of communities in Sfax solar salterns (Tunisia) where viral concentration and virus-to-cell ratios increased along a salinity gradient [11].

In contrast, for other haloarchaeoviruses, such as ΦCh1, the opposite occurs and virus population densities are lowest during high salinity when host populations are high, and highest during low salinity when host populations are low [51]. His1 and S5100 engage in persistent infection when salinities are above the optimum for their hosts, and lyse host cells when salinity is low [49,62]. This may be a strategy for viruses to exit the hosts in order to avoid becoming decimated when host cells are stressed and die [111]. Alternatively, viruses may recognize and attack hosts that are not likely to contribute genes to future generations, and therefore virus growth and lysis in low salinity would not contribute to selection because the hosts will inevitably die [51].

The interpretations of responses to changes in salinity are relevant to broader considerations of virus-host relationships. It has been proposed that low virulence may contribute to how hosts evolve [148]. Viruses with low virulence would maintain a low density thereby posing less threat to the host, and therefore not prompt hosts to evolve mechanisms of defense. Temperate or persistent infection would enable viruses to grow stably with the host, with increased viral infectivity and lytic growth under conditions when the host will die (e.g., low salinity). Under these conditions, the virus may benefit by limiting the evolution of host defense systems. The host may also benefit by having viruses carry, preserve and transfer its cellular DNA to suitable hosts when the cellular population has reestablished after a period of decline.

## 8. Conclusions

Although it has been almost 40 years since haloarchaeoviruses were discovered, there has been relatively little research compared to bacteriophages and eucaryoviruses, in part due to the difficulty in cultivating haloarchaea. However, from studies so far, a great deal of novelty has been documented about certain haloarchaeoviruses. This includes the unique spindle-shaped morphology, capsid structures unseen in other viruses, and a different mode of host infection that challenges the traditional virus dichotomy of temperate *versus* virulent. In addition, the majority of predicted protein-coding genes have no significant matches in current databases. This opens up the possibility of discovering a whole new arena of protein structure and function.

As there is at least one order of magnitude more viruses than hosts, cellular life has been described as bathing in a virtual sea of viruses [28]. Gene transfer agents that are known to package and transfer random pieces of cellular genomic DNA in bacteria and the archaeon *Methanococcus voltae* [149,150], may function in equivalent ways in haloarachaea. Viral infection and recombination appears to generate the mosaic genomes of haloarchaeoviruses and ViPREs, as well as contributing to the evolution of cellular lineages. By facilitating horizontal gene transfer and recombination, variation is bestowed upon populations, enabling adaptation to environmental changes and continuation of speciation [151]. As haloarchaea are relatively slow-growing but demonstrate high levels of genetic variation, haloarchaeoviruses may play an important role in their continuing evolution.

With the majority of haloarchaeoviruses having persistent infection, transfer of genetic material between host haloarchaea may be facilitated without causing harm to the hosts. Whereas viruses have traditionally been considered to primarily have predator-prey relationships with their hosts, haloarchaoviruses may have more of a mutualistic association with haloarchaea. Moreover, while higher trophic predators such as protozoa tend to have low abundance in hypersaline systems compared to haloarchaea [2,19,20], diversity of *Eucarya* can be high and the eucaryotes can feed on the haloarchaea [8,9,10]. Research-into and modelling-of predator-prey relationships therefore needs to take into account the possible ecological relevance of mutualistic association of haloarchaeoviruses with their hosts, as well as protozoan grazing on hosts.

The fact that viruses have not yet been successfully isolated infecting *Hqr. walsbyi,* an abundant member of many halophilic communities, illustrates a challenge in the field. Much in the field remains to be discovered, especially to improve the depth of knowledge about morphology, host attachment, host entry, replication and assembly. While some research on horizontal gene transfer has been performed, selective pressures on hosts and regulation of host cell densities, the full diversity of haloarchaeoviruses, their ecological roles, and the details of their “love-hate” relationship with their hosts remain to be determined.

Advances in DNA sequencing technologies continue to expand the capacity to perform meta/genomics, as do DNA sequencing and mass spectrometry technologies for meta/transcriptomic and meta/proteomic analyses, thereby offering powerful and cost effective avenues for rapidly learning about virus-host interactions, ecology and evolution. Equally so, physiological and biochemical studies of isolates, and particularly importantly, infection and isolation studies of haloarchaeoviruses, must be undertaken; for example, persistent infection would not have been discovered without the use of laboratory, culture-dependent approaches. Based on progress to date and trajectory of the field, the future of haloarchaeovirus research looks to continue to bloom.
